# Prevalence of anencephaly in Africa: a systematic review and meta-analysis

**DOI:** 10.1038/s41598-021-02966-w

**Published:** 2021-12-09

**Authors:** Mohammed Oumer, Anteneh Ayelign Kibret, Amanuel Girma, Ashenafi Tazebew, Mezgebu Silamsaw

**Affiliations:** 1 Department of Human Anatomy, School of Medicine, College of Medicine and Health Sciences, University of Gondar, Gondar, Amhara Ethiopia; 2 Department of Epidemiology, Institute of Public Health, College of Medicine and Health Sciences, University of Gondar, Gondar, Amhara Ethiopia; 3 Departments of Pediatrics and Child Health, School of Medicine, College of Medicine and Health Sciences, University of Gondar, Gondar, Amhara Ethiopia; 4 Department of Internal Medicine, School of Medicine, College of Medicine and Health Sciences, University of Gondar, Gondar, Amhara Ethiopia

**Keywords:** Anatomy, Medical research

## Abstract

Anencephaly is a severe anomaly of the brain that results from the failure of the cephalic part of the neural tube to close during the fourth week. It occurs at least in one per thousand births and is the major cause of fetal loss and disabilities in newborns. The objective of this review is to determine the birth prevalence of anencephaly in Africa. We identified relevant studies via a search of databases like PubMed Central, PubMed/Medline, Science Direct, Joanna Briggs Institute, African Journals Online, Embase, Google Scholar, Web of Science, and Cochrane Library. After examining the heterogeneity of studies via the Cochran Q test and I^2^ test (and Forest plot for visual inspection), the prevalence of anencephaly was estimated using the random-effect meta-analysis model. Consequently, we carried out subgroup, sensitivity, meta-regression, trim and fill, time-trend, and meta-cumulative analyses. In this systematic review and meta-analysis, the twenty-four studies reported a total of 4,963,266 births. The pooled birth prevalence of anencephaly in Africa was 0.14% (95% CI: 0.12, 0.15%). Higher burden of anencephaly was detected in Ethiopia (0.37%, CI: 0.15, 0.58%), Algeria (0.24%, CI: 0.24, 0.25%), and Eritrea (0.19%, CI: 0.19, 0.19%). The higher pooled prevalence of anencephaly was observed in the studies that included both live births and stillbirths (0.16%) and in studies done after the year 2010 (0.25%) whereas, the lower burden was detected among countries that had a mandatory folic acid fortification (0.05%). High birth prevalence of anencephaly was detected in Africa. Strong prevention and control measures should be the priority because of an increment in the magnitude of anencephaly. Helping in prevention programs, which should be the ultimate contribution of this study to the field.

## Introduction

Anencephaly is a severe defect of the brain that results from failure of the rostral neuropore to close during the fourth week of development^1–8^. As a result, the vault of the cranium does not form, leaving the malformed neural tissue exposed. Later, this tissue degenerates, leaving a mass of necrotic tissue due to the abnormal structure and vascularization of the embryonic exencephalic neural tissue^1–4^. Sometimes, the closure abnormality of the tube extends caudally into the spinal cord and the malformation is known as craniorachischisis^2,3^.

Anencephaly, a common lethal anomaly, occurs in 1.0–4.7 per 1000 births and is more common in females (female: male ratio of 4:1) than in males^1,3,4^. It is the most common serious anomaly seen in stillborn fetuses. Newborns with this severe defect may survive after birth, but only for a short period^1,4^. Newborns delivered with anencephaly show persistent unconsciousness due to lack of functioning cerebral cortex and varying degrees of brain stem functions causing brain death^4^.

Anencephaly is always related to the absence of the calvaria^1^. An ossification abnormality in the bones of the cranium results in meningocele, meningoencephalocele, and meningohydroencephalocele^2,5–7^. The squamous part of the occipital bone is the most common malformed bone. Only meninges bulge through a small occipital bone opening, which is known as meningocele, but the part of the brain and part of the ventricle are protruding through the large defect in the skull, the defects are called meningoencephalocele and meningohydroencephalocele, respectively^8–14^. These malformations occur one in every two thousand births^2,3^.

It is the major cause of fetal loss and disabilities in newborns and it is considered a significant public health problem^4–6,15,16^. It is associated with substantial mortality, morbidity, and psychological costs^6–8^.

Anencephaly is suspected in utero when there is an elevated level of alpha-fetoprotein in the amniotic fluid. The abnormality can be diagnosed by ultrasonography and magnetic resonance imaging because extensive parts of the brain and the vault of the skull are absent^1–3^. Because anencephalic fetuses lack a swallowing reflex in the presence of a large defect (the fetus lacks the neural control for swallowing amniotic fluid, thus, the fluid does not pass into the intestines for absorption and subsequent transfer to the placenta for disposal), the last two months of pregnancy are characterized by polyhydramnios (excess amniotic fluid)^1,3^.

Anencephaly, although it is preventable, is still the major cause of death and newborn disability in the world. It can be prevented by having women take 400 µg of folic acid per day before and during pregnancy^2,3,17,18^.

This review is helpful to dig out the burden of anencephaly in Africa and provide valuable information to the government, policymakers, health professionals (Anatomists, Pediatricians, Medical students, for instance), researchers, communities, and Non-Governmental Organizations to play a role in reducing the burden, and making further research towards anencephaly. Furthermore, little is known about the magnitude of anencephaly in the region as a whole. Therefore, the aim of the present systematic review and meta-analysis is to determine the pooled birth prevalence of anencephaly in Africa.

## Results

**Figure 1:**
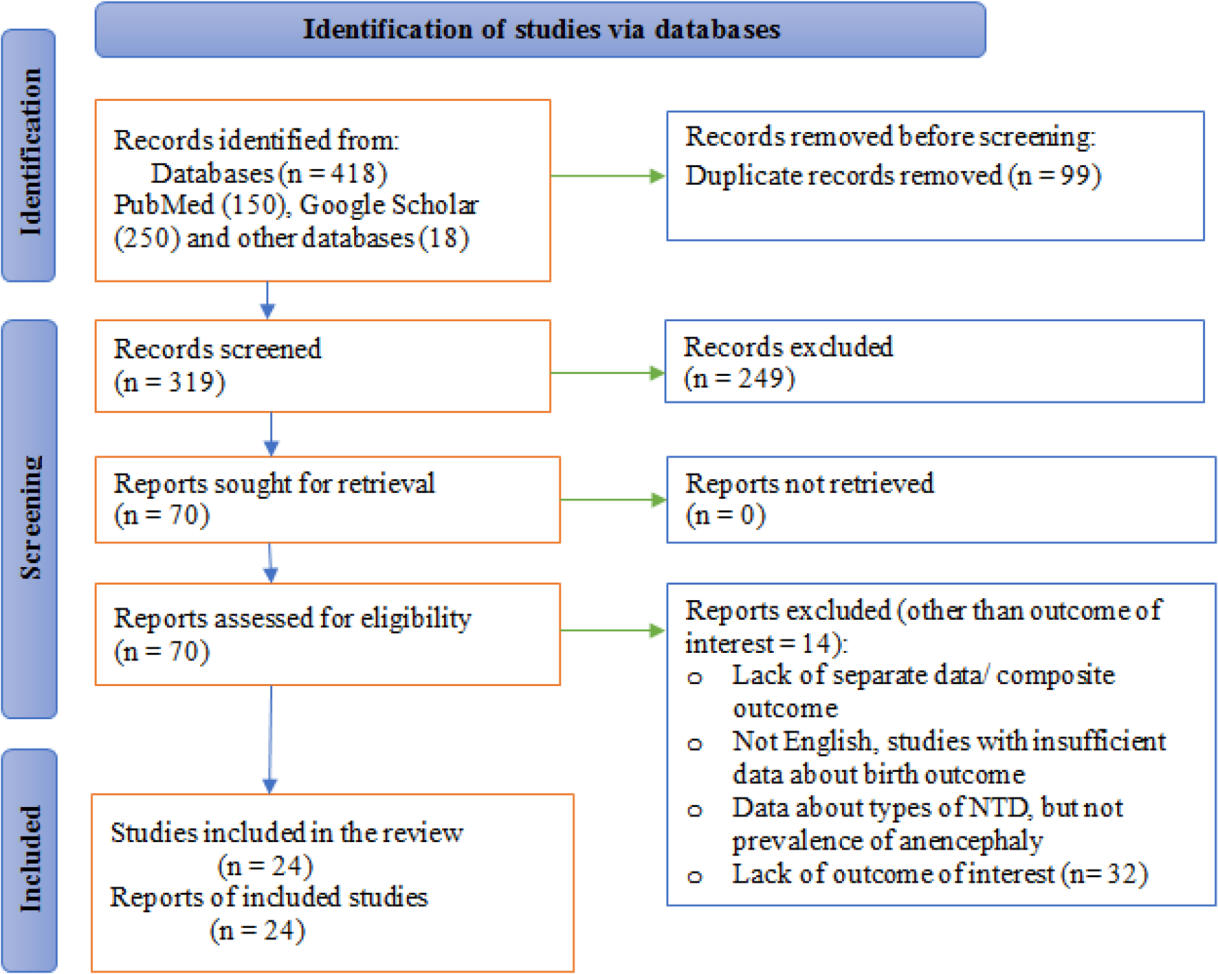
Study selection flow diagram based on the PRISMA 2020 statement: an updated guideline for reporting systematic reviews^19^.

The comprehensive search of databases yielded four hundred eighteen studies about anencephaly in Africa. Of these, 99 were excluded because of duplication. Of the rest articles (319), 249 articles were excluded after reviewing the titles and abstracts. Full texts of the remaining 70 articles were screened and assessed for eligibility. Thus, twenty-four studies have fulfilled the inclusion criteria and have been included in the systematic review and meta-analysis (Fig. 1).

In this systematic review, twenty-four studies reported 4,963,266 births. All included studies were cross-sectional (n = 17) and prospective cohort study design (n = 7)^5–14,20–33^. Studies conducted in Ethiopia (n = 5)^5,9–11,14^, Nigeria (n = 4)^7,12,22,23^, South Africa (n = 3)^27,30,31^, Algeria (n = 2)^8,28^, Sudan (n = 2)^21,33^, Tunisia, Eritrea, Democratic Republic (DR) Congo, Libya, Egypt, Ghana, Tanzania, and Cameron^6,13,20,24–26,29,32^ were identified (Table 1).

**Table 1:** The characteristics of original studies included in the systematic review and meta-analysis, 2020.

First author	Year	Country	Study design	Sample size	Duration (months)	Prevalence (%)
Gedefaw et al.^5^	2018	Ethiopia	Cross-sectional	8677	7	0.78
Nasri et al.^6^	2014	Tunisia	Cross-sectional*	3,803,889	240	0.005
Anyanwu et al.^7^	2015	Nigeria	Cross-sectional	1456	9	0.07
Houchar et al.^8^	2008	Algeria	Cross-sectional*	28,500	36	0.24
Berihu et al.^9^	2018	Ethiopia	Cross-sectional	14,903	9	0.66
Taye et al.^10^	2019	Ethiopia	Cross-sectional	76,201	6	0.05
Abebe et al.^11^	2020	Ethiopia	Cross-sectional	45,951	60	0.14
Nnadi et al.^12^	2016	Nigeria	Prospective	10,163	36	0.05
Estifanos et al.^13^	2017	Eritrea	Cross-sectional*	39,803	24	0.19
Legesse et al.^14^	2019	Ethiopia	Prospective	956	7	0.21
Ahuka et al.^20^	2006	DR Congo	Cross-sectional*	8824	96	0.011
Oumer et al.^21^	2016	Sudan	Cross-sectional	36,785	12	0.049
Alrede et al.^22^	1992	Nigeria	Prospective	5977	36	0.033
Ekanem et al.^23^	2008	Nigeria	Cross-sectional*	127,929	276	0.016
Singh et al.^24^	2000	Libya	Prospective	15,938	12	0.075
Mohammed et al.^25^	2011	Egypt	Cross-sectional	5000	7	0.02
Njamnshi et al.^26^	2008	Cameron	Cross-sectional*	52,710	120	0.008
Sayed et al.^27^	2008	South Africa	Prospective	53,000	9	0.037
Houcher et al.^28^	2012	Algeria	Cross-sectional*	28,500	36	0.242
Anyebuno et al.^29^	1993	Ghana	Cross-sectional*	19,094	24	0.084
Venter et al.^30^	1995	South Africa	Prospective	10,380	40	0.171
Buccimazza et al.^31^	1994	South Africa	Cross-sectional*	516,252	240	0.032
Kinasha et al.^32^	2003	Tanzania	Cross-sectional*	34,000	24	0.012
Elsheikh et al.^33^	2009	Sudan	Prospective	18,378	12	0.125

Key: *Cross-sectional study design with a retrospective review.

Surprisingly, all studies included were facility-based studies. Currently, Ethiopia has voluntary folic acid fortification whereas South Africa, Nigeria, Ghana, and Cameron have mandatory folic acid fortification with Wheat Flour, Maize Flour, and Rice. Studies considered after the implementation of mandatory folic acid fortification policy were South Africa and Nigeria (Table 2).

**Table 2:** The summary of studies included in the systematic review and meta-analysis, 2020.

First author	Setting	Prevalence period	Folic acid fortification policy	Birth outcome, denominator
Gedefaw et al	Hospital-based	Feb 2016–Aug 2016	Yes*	LB + SB
Nasri et al	Hospital-based	1991–2011	No	LB + SB
Anyanwu et al	Hospital-based	Apr 2013–Dec 2013	Yes	LB
Houchar et al	Hospital-based	2004–2006	No	LB + SB
Berihu et al	Hospital-based	Oct 2016–Jun 2017	Yes*	LB + SB
Taye et al	Hospital-based	Jan 2015–Jul 2015	Yes*	LB
Abebe et al	Hospital-based	Sep 2011–Dec 2015	Yes*	LB + SB
Nnadi et al	Hospital-based	Jan 2011–Dec 2013	Yes	LB + SB
Estifanos et al	Hospital-based	Jan 2007–Aug 2011	No	LB + SB
Legesse et al	Hospital-based	Oct 2018–Apr 2019	Yes*	LB + SB
Ahuka et al	Hospital-based	Jan 1993–Aug 2001	No	LB
Oumer et al	Hospital-based	Aug 2014–Jul 2015	No	LB + SB
Alrede et al	Hospital-based	Jun 1987–Jun 1990	No	LB + SB
Ekanem et al	Hospital-based	1980–2003	No	LB + SB
Singh et al	Hospital-based	1995–1996	No	LB + SB
Mohammed et al	Hospital-based	Mar 2007–Oct 2007	No	LB
Njamnshi et al	Hospital-based	Jun 1997–Dec 2006	No	LB + SB
Sayed et al	Hospital-based	Oct 2004–Jun 2005	Yes	LB + SB
Houcher et al	Hospital-based	2010–2012	No	LB + SB
Anyebuno et al	Hospital-based	Jan 1991–Dec 1992	No	LB + SB
Venter et al	Hospital-based	Jun 1989–Dec 1992	No	LB
Buccimazza et al	Hospital-based	Jan 1973–Dec 1992	No	LB + SB
Kinasha et al	Hospital-based	Jan 2000–Jan 2002	No	LB
Elsheikh et al	Hospital-based	Feb 2003–Jan 2004	No	LB + SB

Key: LB: Live births; SB: Stillbirths; *: Voluntary folic acid fortification.

Each study was evaluated using the JBI critical appraisal checklist for prevalence studies, it has nine questions/items with options of Yes, No, Unclear, or Not Applicable (Table 3). The quality assessment grading for all items was based on the JBI descriptions for each item (methodological guidance for systematic reviews of epidemiological studies reporting the prevalence data). The quality score of each study was described in Table 3^5–14,20–33^.

**Table 3:** The quality status of studies based on JBI critical appraisal checklist for studies reporting prevalence data, 2020.

Studies	Appropriate sampling frame?	Appropriate sampling?	Adequate sample size?	Detail setting description?	Analysis with sufficient coverage?	Valid method to identify the condition?	Reliable measurement?	Appropriate statistical analysis?	Adequate response rate?	Total, out of 9
Gedefaw et al	Yes	Yes	Yes	Yes	Yes	Yes	Yes	Yes	N/A	9
Nasri et al	Yes	N/A	Yes	Yes	Yes	UC	UC	Yes	N/A	7
Anyanwu et al	N/A	N/A	Yes	Yes	Yes	Yes	Yes	Yes	N/A	9
Houchar et al	Yes	N/A	Yes	UC	Yes	No	No	Yes	N/A	6
Berihu et al	N/A	Yes	Yes	Yes	Yes	Yes	Yes	Yes	Yes	9
Taye et al	N/A	N/A	Yes	Yes	Yes	No	Yes	Yes	Yes	8
Abebe et al	Yes	N/A	Yes	Yes	Yes	No	Yes	Yes	N/A	8
Nnadi et al	Yes	N/A	Yes	Yes	Yes	Yes	Yes	Yes	N/A	9
Estifanos et al	Yes	N/A	Yes	No	No	Yes	No	Yes	No	5
Legesse et al	Yes	No	Yes	Yes	No	No	Yes	Yes	No	5
Ahuka et al	Yes	N/A	Yes	Yes	Yes	Yes	UC	UC	N/A	7
Oumer et al	Yes	N/A	Yes	Yes	Yes	Yes	UC	Yes	N/A	8
Alrede et al	Yes	N/A	Yes	UC	UC	UC	UC	Yes	N/A	5
Ekanem et al	Yes	N/A	Yes	No	Yes	UC	No	Yes	N/A	6
Singh et al	Yes	N/A	Yes	Yes	Yes	Yes	UC	Yes	N/A	8
Mohammedetal	N/A	N/A	Yes	Yes	Yes	Yes	UC	Yes	N/A	8
Njamnshi et al	Yes	N/A	Yes	Yes	Yes	UC	No	Yes	N/A	7
Sayed et al	Yes	N/A	Yes	No	Yes	UC	No	Yes	N/A	6
Houcher et al	Yes	N/A	Yes	UC	Yes	UC	No	Yes	N/A	6
Anyebuno et al	Yes	N/A	Yes	Yes	Yes	UC	No	Yes	N/A	7
Venter et al	Yes	N/A	Yes	Yes	Yes	Yes	UC	Yes	N/A	8
Buccimazza etal	Yes	N/A	Yes	Yes	Yes	UC	No	UC	N/A	6
Kinasha et al	Yes	N/A	Yes	No	Yes	UC	No	Yes	N/A	6
Elsheikh et al	N/A	N/A	Yes	No	UC	UC	No	Yes	N/A	5

Key: UC: Unclear, N/A: Not Applicable. N/A for appropriate sampling means the study included all participants rather than sampling methods; N/A for adequate response rate means the study considered all recorded cases from all participants, so it is adequate; UC means it may be considered but not explicitly stated in the manuscript. For adequate sample size, as all participants were included in the study during the study period, we considered an adequate sample size even if they did not calculate sample size (it is a total coverage).

### Meta-analyses

**Figure 2:**
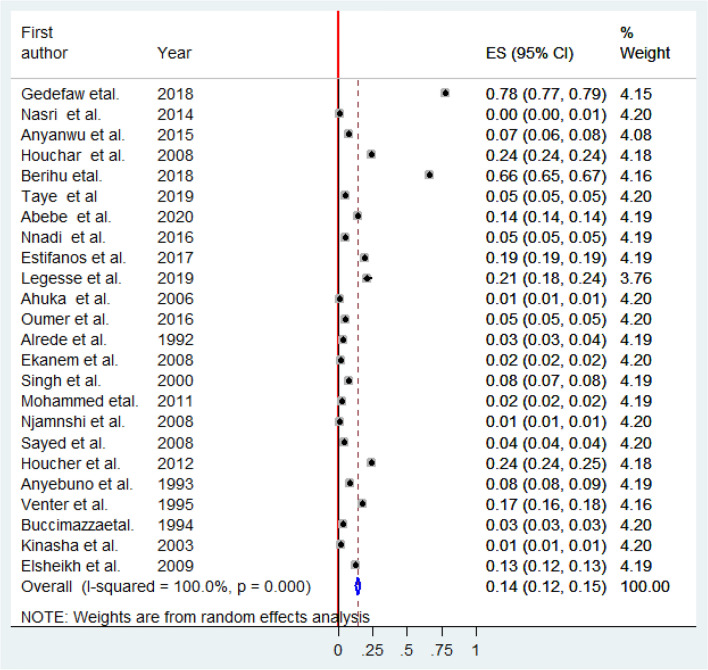
Forest plot showing the pooled prevalence of anencephaly in Africa, 2020.

In the present meta-analysis, the pooled birth prevalence of anencephaly was 0.14% or 1.4 per 1000 births (95% CI: 0.12, 0.15%) (Fig. 2). For all studies^5–14,20–33^, the median (per 100) value of birth anencephaly was 0.06% and the inter-quartile range was between 0.03 and 0.18. The minimum and maximum values of birth anencephaly were 0.005 and 0.78%, respectively.

Meta-analyses were performed to estimate the birth prevalence of anencephaly based on the study country, study design, birth outcome, period prevalence, and folic acid fortification status. High pooled prevalence of anencephaly was detected in Ethiopia 0.37% (95% CI: 0.15, 0.58%), Algeria 0.24% (CI: 0.24, 0.25%), and Eritrea 0.19% (CI: 0.19, 0.19%) (Table 4).

**Table 4:** The pooled prevalence of anencephaly among African countries, 2020.

S. No	Country	Prevalence of anencephaly % (95% CI)
1	Ethiopia	0.37 (0.15, 0.58)
2	Tunisia	0.005 (0.005, 0.005)
3	Nigeria	0.04 (0.02, 0.06)
4	Algeria	0.24 (0.24, 0.25)
5	Eritrea	0.19 (0.19, 0.19)
6	DR of Congo	0.01 (0.009, 0.01)
7	Sudan	0.09 (0.01, 0.16)
8	Libya	0.08 (0.07, 0.08)
9	Egypt	0.02 (0.016, 0.02)
10	Cameron	0.008 (0.007, 0.009)
11	South Africa	0.079 (0.05, 0.11)
12	Ghana	0.084 (0.08, 0.09)
13	Tanzania	0.01 (0.01, 0.013)
Total	D + L pooled	0.14 (0.12, 0.15)

The Der Simonian and Laird’s (D + L) method was considered for significant heterogeneity between countries (*P* < 0.001, I^2^ = 99–100%).

The pooled birth prevalence of anencephaly for cross-sectional was 0.15% (95% CI: 0.13, 0.17%) and for prospective cohort design was 0.10% (95% CI: 0.07, 0.13%) (*P* value < 0.001, I^2^ = 99.8–100%).

**Figure 3:**
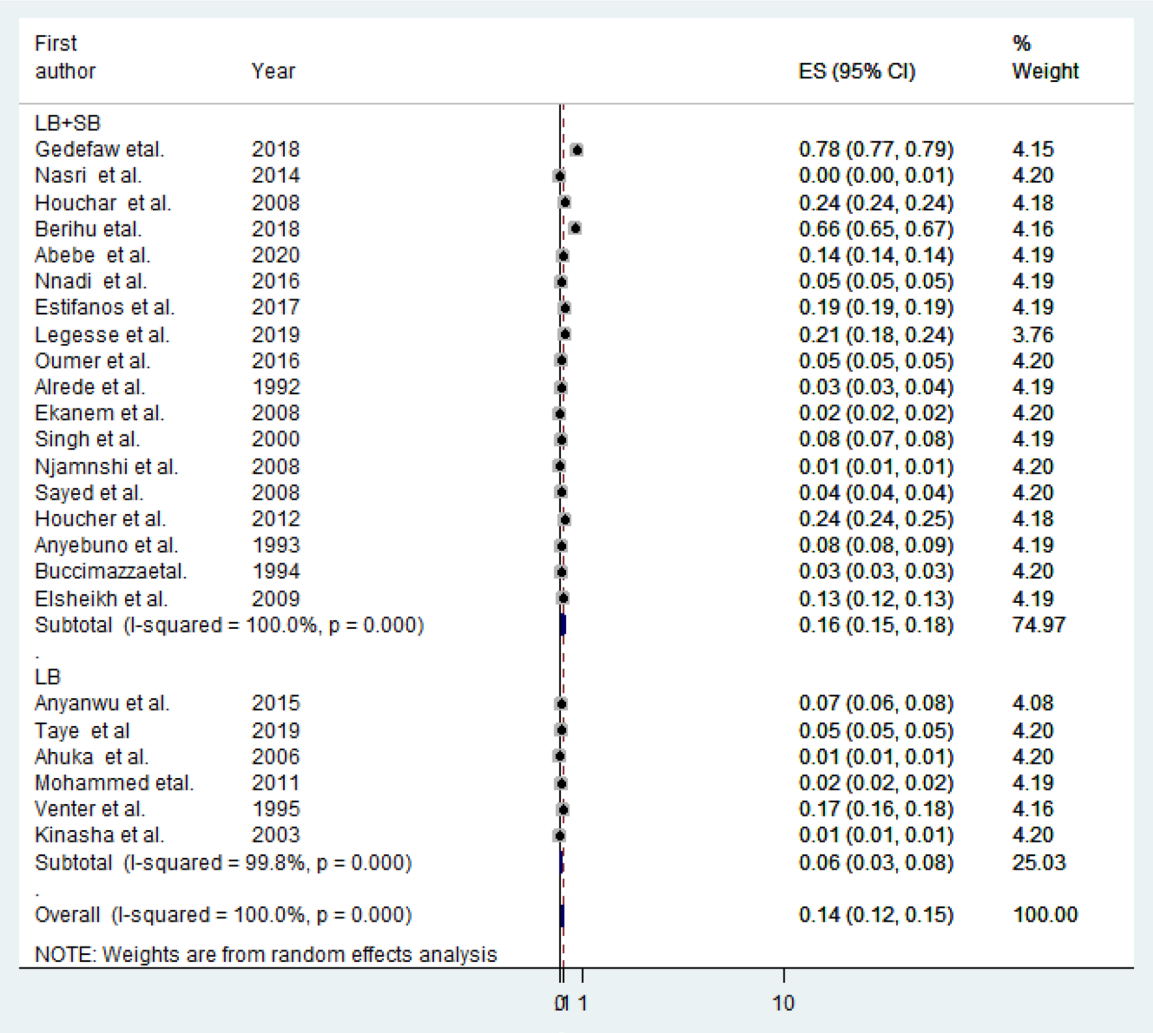
Sub-group analysis showing the pooled prevalence of anencephaly based on birth outcome (live births only (LB), live births and stillbirths (LB + SB)) in Africa, 2020.

The pooled birth prevalence of anencephaly for live births (LB) only (contain six studies) was 0.06% (95% CI: 0.03, 0.08%) and for both live birth and stillbirths (LB + SB) (contain eighteen studies) was 0.16% (95% CI: 0.15, 0.18%) (Fig. 3).

**Figure 4:**
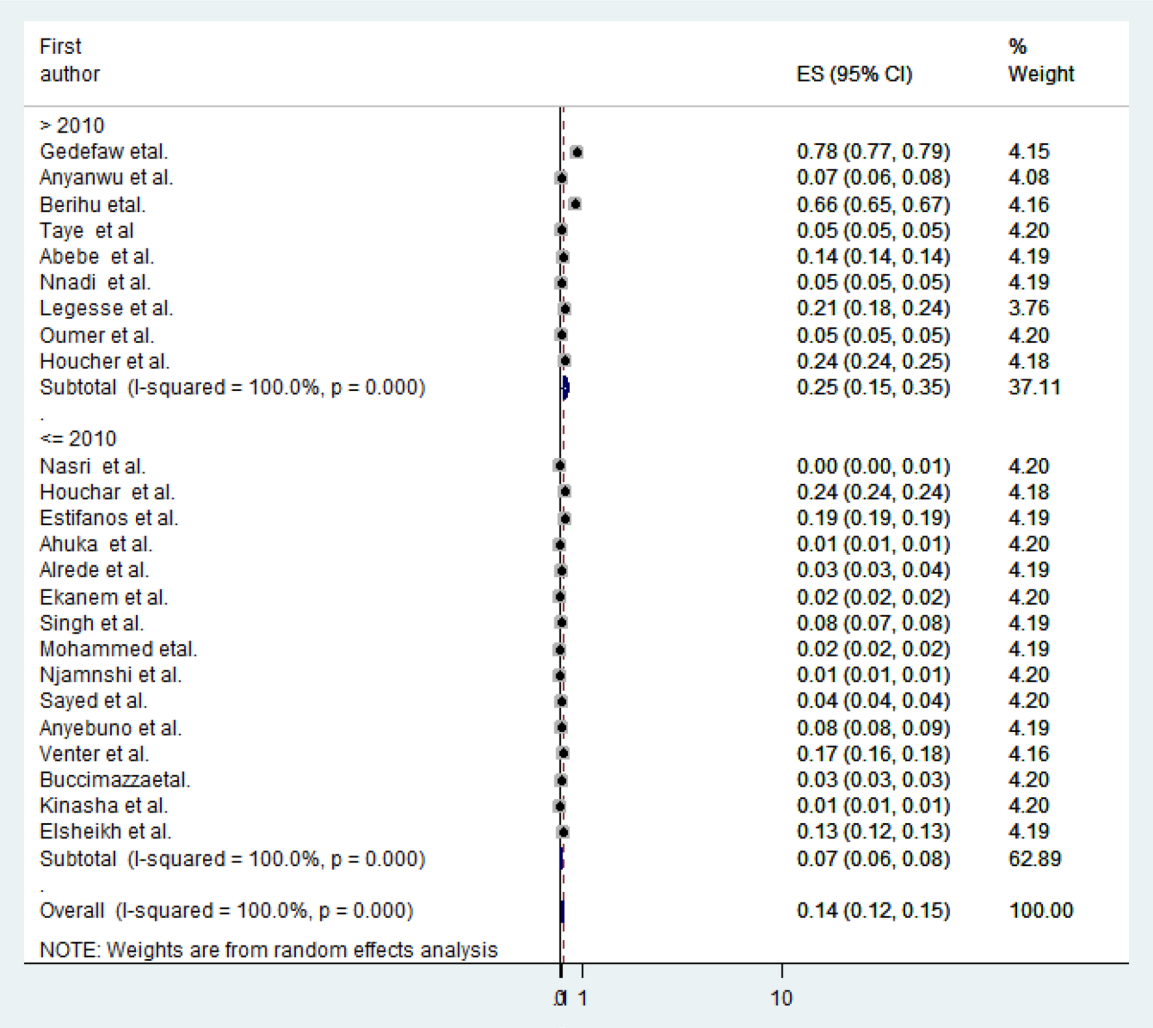
Sub-group analysis showing the pooled prevalence of anencephaly based on period prevalence in Africa, 2020.

Considering two prevalence periods (> 2010 and ≤ 2010 years), the pooled prevalence of anencephaly for studies done after 2010 (contain nine studies) was 0.25% (95% CI: 0.15, 0.35%) and for studies done before 2010 (contain fifteen studies) was 0.07% (95% CI: 0.06, 0.08%) (Fig. 4).

Even if their publication year is after the year 2010 (Nasri et al., Estifanos et al., and Mohammed et al.), most of their study period were found before 2010, so we categorized them as studies done before 2010. Based on four periods, the pooled prevalence of anencephaly for the period after 2010 (contain nine studies) was 0.25% (95% CI: 0.15, 0.35%), for the period between 1991 and 2011 was 0.0% (only one study done for 20 years), for the period between 2000 and 2010 (contain seven studies) was 0.09% (95% CI: 0.05, 0.13%), and for the period before 2000 (contain seven studies) was 0.06% (95% CI: 0.05, 0.07%). Based on folic acid fortification policy (*P* value < 0.001, I^2^ = 100%), the pooled birth prevalence of anencephaly for countries that had a mandatory folic acid fortification (like Nigeria and South Africa) was lower, 0.05% (95% CI: 0.04, 0.06%), than for those free of a mandatory folic acid fortification policy, 0.15% (95% CI: 0.13, 0.17%).

Using meta-regression analysis (multivariable), sample size (B-Coefficient = − 9.14e^−09^, *P* value = 0.888), year of publication (B-Coefficient =  − 0.004, *P* value = 0.589), duration of the study in month (B-Coefficient =  − 0.001, *P* value = 0.117), the JBI quality score (B-Coefficient =  − 0.031, *P* value = 0.509), study country (B-Coefficient =  − 0.012, *P* value = 0.508), study design (B-Coefficient =  − 0.195, *P* value = 0.110), folic acid fortification status (B-Coefficient =  − 0.151, *P* value = 0.290), outcome status (B-Coefficient =  − 0.176, *P* value = 0.103), and period prevalence (B-Coefficient = 0.016, *P* value = 0.915) were analyzed for the source of heterogeneity. None of them was statistically significant. Nevertheless, at bivariate analysis, study country (B-Coefficient =  − 0.021, *P* value = 0.038), folic acid fortification status (B-Coefficient =  − 0.167, *P* value = 0.045), and period prevalence (B-Coefficient =  − 0.179, *P* value = 0.026) were significant at below 0.05.

**Figure 5:**
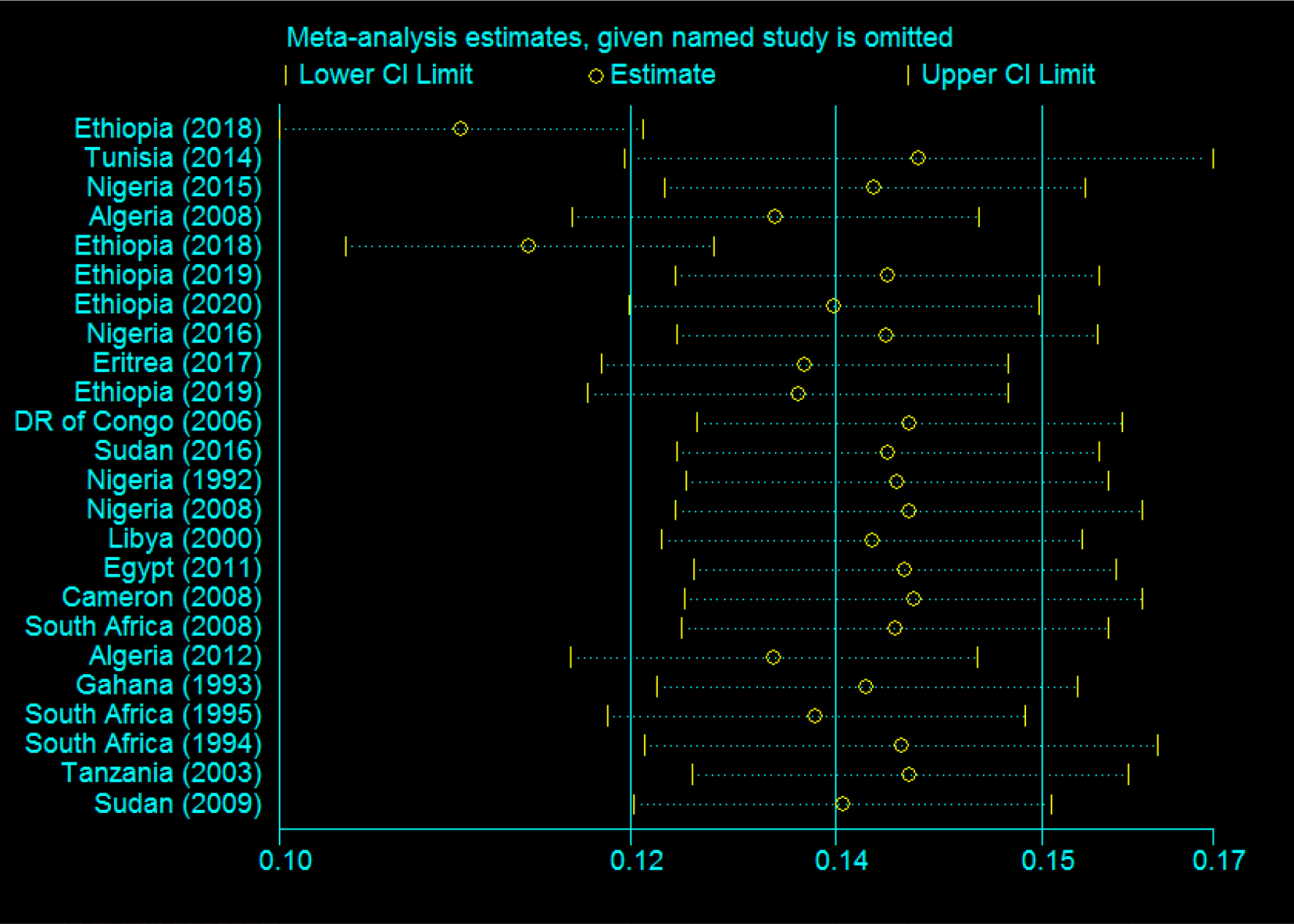
Sensitivity analysis to see the influence of each study in Africa, 2020.

In this review, most of the studies had a uniform influence on the overall estimation of meta-analysis except two studies conducted in Ethiopia in 2018, which had some influence over other studies^5,9^ (Fig. 5).

**Figure 6:**
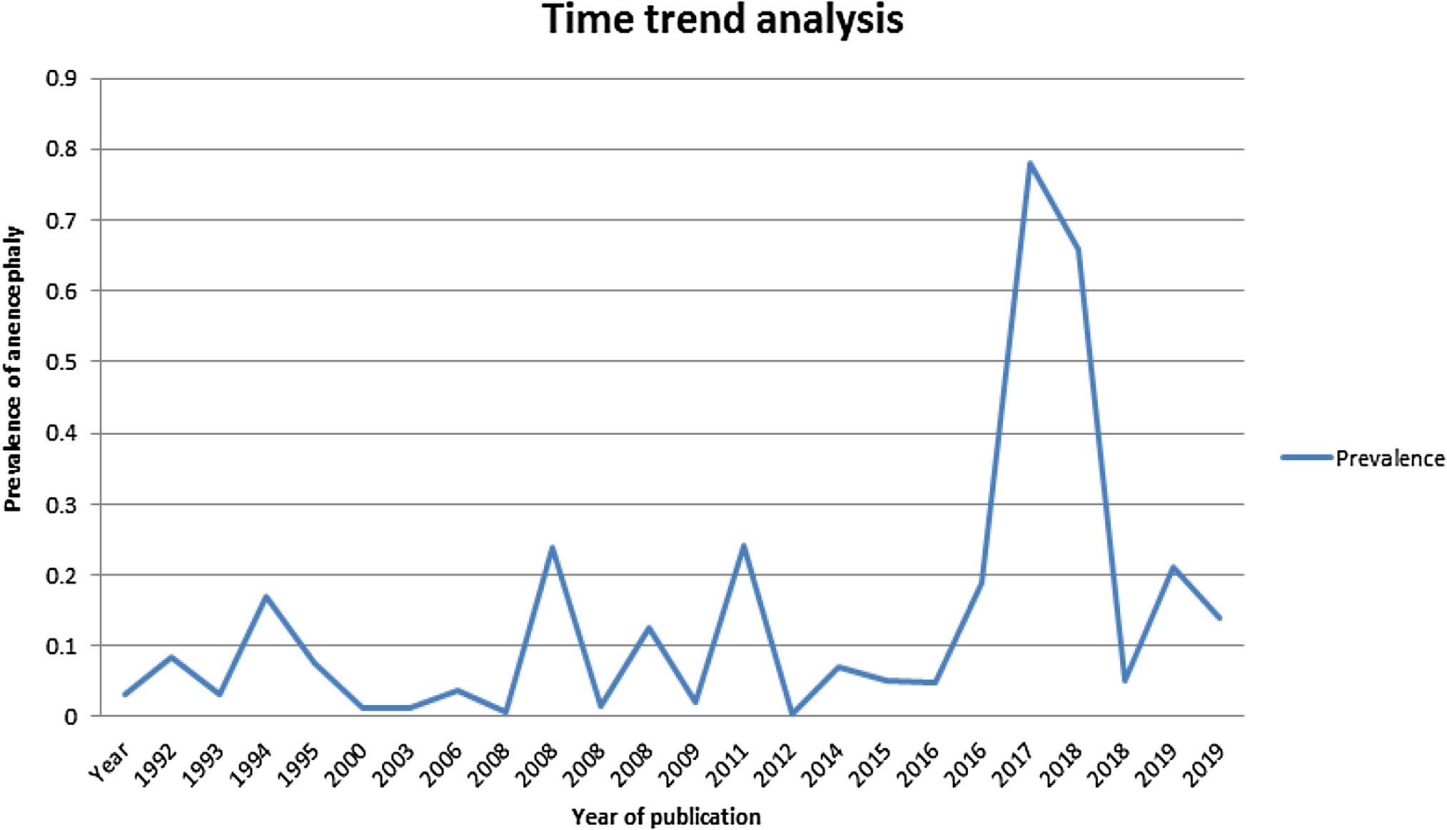
Time trend analysis of the prevalence of anencephaly in relation to publication year in Africa, 2020.

The time trend analysis displayed the relationship between the birth prevalence of anencephaly and publication year from 1992 (0.03%) to 2020 (0.14%). In this trend in Africa, the highest peak of anencephaly in prevalence was observed between 2016 and 2018 (Fig. 6).

**Figure 7:**
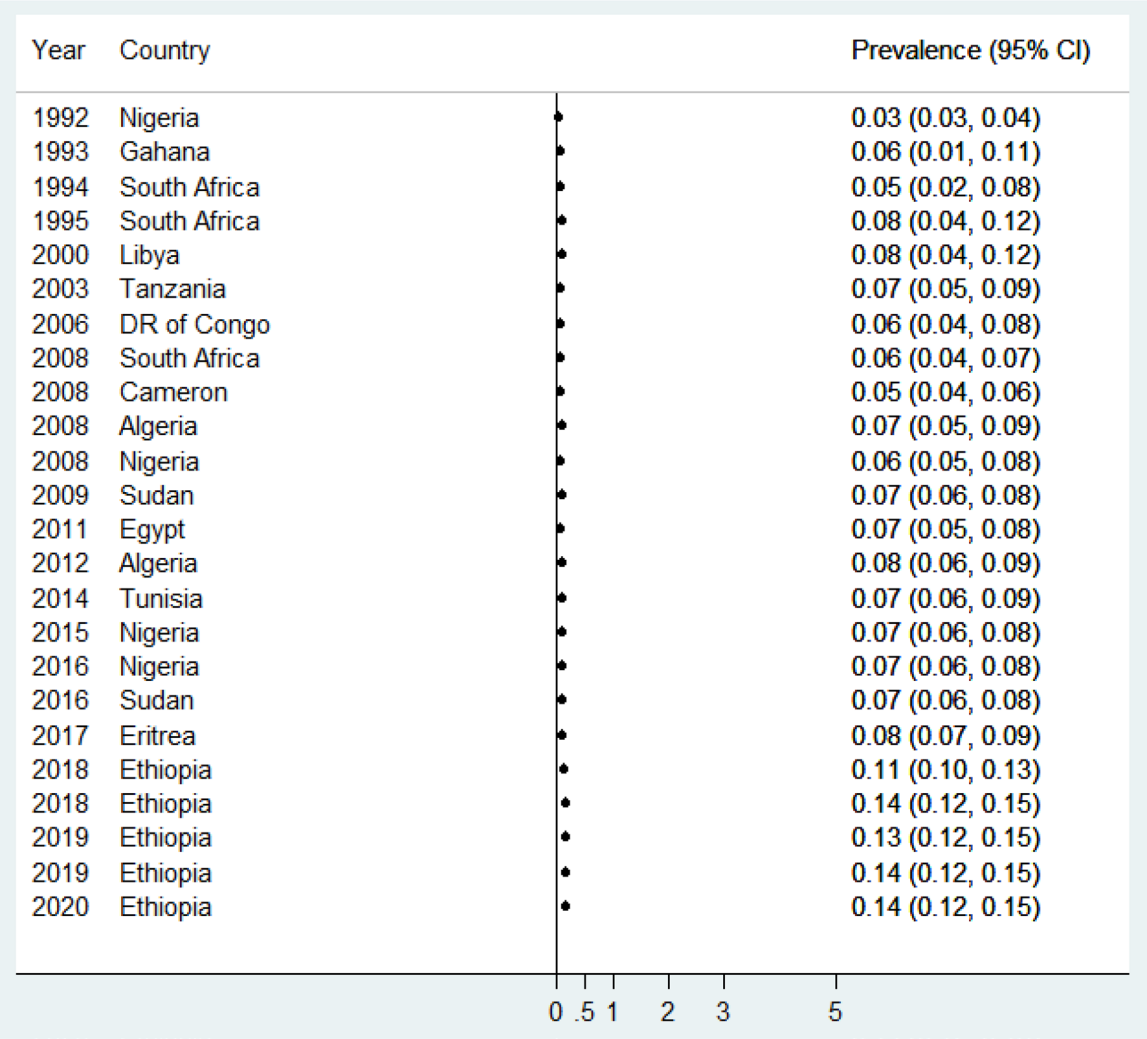
Meta-cumulative analysis of the prevalence of anencephaly in Africa, 2020.

The present review described the cumulative effect of the birth prevalence of anencephaly from the year 1992 (0.03%) to the year 2020 (0.14%) (Fig. 7).

**Figure 8:**
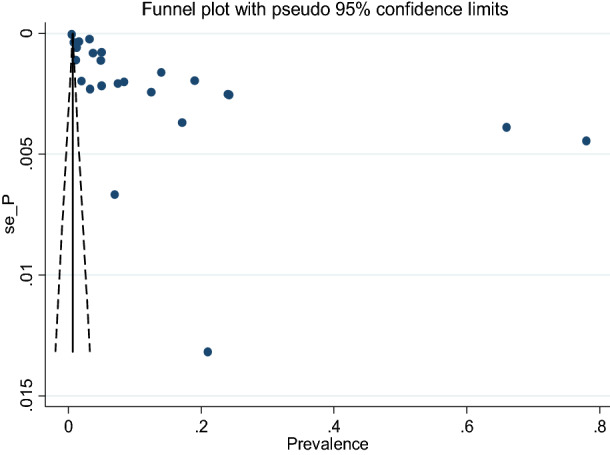
Funnel’s publication bias plot, 2020.

Based on the Funnel plot, considerable publication bias was observed (Fig. 8).

**Figure 9:**
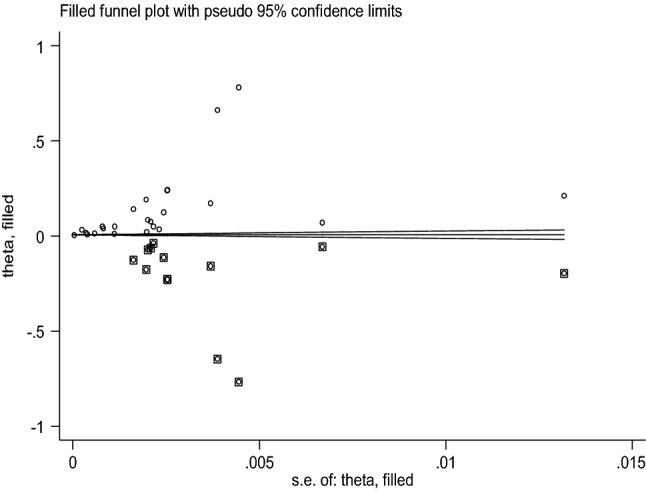
Trim and fill analysis of funnel plot to trim publication bias among studies, 2020.

As a result, we conducted the trim and fill meta-analyses to adjust this bias. We analyzed thirty-seven studies (thirteen studies were filled) in the fill meta-analyses and the pooled birth prevalence of anencephaly using the random-effect model was 0.012% (95% CI: − 0.005, 0.03%) (Fig. 9). This adjusted estimate suggested a lower risk of bias than the original analysis.

## Discussion

The present systematic review and meta-analysis were carried out to determine the pooled birth prevalence of anencephaly in Africa. The burden of anencephaly seems to become decreasing due to different preventive measures and folic acid fortifications in high-income countries. In Africa, the hidden burden of anencephaly was very high and the primary data research, as well as systematic review and meta-analysis that show this burden, were so scarce. Even in the global estimation of the burden, the inclusion of studies from Africa was a few to represent the whole continent due to different reasons. Nevertheless, the effects of the anomaly correlate with mortality, disability, and psychological costs. It is an important public health problem that can be prevented with folic acid supplementation and fortification of staple foods^1–3,17,18,34,35^. Thus, the evidence provided a birth prevalence estimate in Africa. The pooled birth prevalence of anencephaly was 0.14%, ranging from 0.12% to 0.15% (or 1.4 per 1000 births). This finding is comparable with the review conducted globally that estimates the live birth prevalence of anencephaly was 0.03% with the range of 0.008 to 0.11%^34^. In addition, the review reported an estimate based on all pregnancies resulting in live births, stillbirths, and terminations were 0.103% for anencephaly. This review result suggested that low-and middle-income countries were mostly affected by this anomaly each year^34^. However, the review did not include studies from Africa except for two studies, and representativeness for Africa has decreased for this reason. A systematic review in India reported the prevalence of anencephaly, which was 0.21%, ranging from 0.16 to 0.28%^15^. Our finding was supported by the previous evidence that indicated anencephaly is a common lethal anomaly, occurring at least once in every thousand births^1–3,35^. Recent evidence proves that there is variation in the prevalence of anencephaly in time, place, and population to population^16^. In different times and regions, the prevalence of anencephaly ranges from one in every thousand births to one in every five thousand births. Income level and instituted folic acid fortification are the main factors that determine the prevalence of anencephaly in one country. A significant difference in the birth prevalence of anencephaly across countries was detected in this review. A very high (0.37%) birth prevalence of anencephaly was detected in Ethiopia for example, the prevalence in Algeria was 0.24%, and in Eritrea was 0.19%. The lowest prevalence was detected in Tunisia (0.005%). Maybe this difference among countries comes due to the levels of knowledge of mothers about folic acid supplementation, the country’s health policies regarding folic acid fortification, and other preventive measures. Furthermore, the difference in prevalence estimate among countries may be due to the difference in the presence of robust surveillance systems that track all pregnancy outcomes (some studies may use a good surveillance and tracking system but others may not have tracked all births). These variations may underestimate the overall burden of anencephaly in Africa. The prevalence estimates were also affected by the difference of countries having access to antenatal screening and terminations. If the terminated cases of anencephaly during pregnancy are very high in one country, it is less likely to have high prevalence estimates at birth. Studies reported that the presence of wide geographical variation in prevalence estimates between the countries^16^. In Tunisia, the lowest prevalence may be related to the detection methods used, reporting and recording system, and the issue of terminated cases. Moreover, the adequacy of the sample size (extremely high sample size in Tunisian study) may affect the estimated report. In the present review, time trend analysis displayed that there was variation in different periods. The highest burden of anencephaly was observed between 2016 and 2018. This finding was also supported by a subgroup analysis of the period prevalence (high prevalence was detected after the year 2010). The increment in the prevalence of anencephaly in this duration may be due to a change in detection methods, an increment of the practices in documenting and reporting cases, an increase of the demands for fetal pathological examinations over these years, or a real increase in disease. The prevalence estimate in live births (0.06%) was lower than estimates from both live births and stillbirths (0.16%). Besides, the prevalence estimate is affected by the status of a mandatory folic acid fortification policy. Because all studies in the current review were hospital-based studies, an underestimation of the prevalence estimates should be noted, as the review did not consider many stillbirths and home births that are delivered in the community setting.

This review finding will help to improve the prevention and control programs in African countries. The severity of the defect, the observed differences in prevalence estimate among countries, may inform to modify the clinical and policy guidelines in the prioritization of interventions in Africa. It is remarkable if the next step will be that all African countries should adopt and implement a mandatory folic acid fortification policy. Besides, there should be started/improved robust surveillance systems in each country to track all pregnancy outcomes, particularly birth defects. Notably, this review highlight the birth prevalence of anencephaly in African countries, providing crucial evidence for policymakers, clinicians, and the concerned bodies who neglected the burden of this defect. The high burden detected in this review may initiate the policymakers to apply effective prevention strategies and may use their ultimate potential in reducing the burden of the anencephaly and making further research possible (additional clinical studies to focus on risk factors, prevention, intervention, and psychosocial outcomes of the defect in isolated form).

As many strengths of the review as it is, the outputs of the present review should be interpreted based on some limitations; the estimate did not include the terminated pregnancy/cases of anencephaly and this may decrease the prevalence estimates. Furthermore, the variability of the sample size in the included studies might influence the pooled birth prevalence estimates. Besides, the presence of significant variation across countries may underestimate the overall burden in Africa. Further, English-written articles were included in order to meticulously examine the quality of the articles. The review represented the studies from the twenty-four African countries due to limited available data about anencephaly.

## Conclusions

The present systematic review and meta-analysis showed that a high birth prevalence of anencephaly was detected in Africa.

The birth-pooled prevalence of anencephaly was very high in Ethiopia and Algeria.

The higher pooled prevalence of anencephaly was observed in the studies included both live births and stillbirths and in studies done after the year 2010 whereas, the lower burden of anencephaly was detected among countries that had a mandatory folic acid fortification.

Therefore, we would like to inform policymakers that the pooled birth prevalence estimates are possible underestimates due to the lack of robust surveillance systems in Africa. The meta-analysis should not impact policy decisions on folic acid-based prevention efforts in Africa negatively where policymakers may feel that this is not a big enough problem for prioritizing prevention funds. Moreover, strong prevention and control measures should be the priority because of an increment in the magnitude of anencephaly, and limited available data on anencephaly in Africa informs the need for additional primary research that would improve the estimated prevalence of anencephaly and recommend favorable aid policies through maternal education on preventive measures. Helping in prevention programs, which should be the ultimate contribution of this study to the field.

## Methods

### Review outcome, reporting of the findings, and searching strategies

The outcome of the present review was the birth prevalence of anencephaly in Africa. Birth prevalence of anencephaly is defined as the number of anencephaly cases of live births and/or stillbirths at birth, after 28 weeks of gestation, (numerator) from the total number of births (live births and/or stillbirths) during the study period (denominator). Remarkably, we performed this review in accordance with the PRISMA 2020 (preferred reporting items for systematic reviews and meta-analysis) guidelines^19^ (Supplementary file 1). The PROSPERO registered the protocol of this review with a registration ID of CRD42021229940. Initially, to avoid duplication, we checked the unavailability of the systematic review and meta-analysis on the topic of interest in the JBI (Joanna Briggs Institute) Library, Cochrane Library, and DARE database. Then, we identified relevant studies via a search of databases like PubMed Central, PubMed/Medline, Science Direct, Web of Science, Embase, JBI Library, African Journals Online, WHO, CINAHL, UCSF, Scopus, and Cochrane Library up to September 14, 2020. Grey literature was retrieved using Google and advanced Google Scholar searches. Moreover, we navigated the reference lists of identified articles for additional eligible studies. The primary search was carried out in an advanced PubMed database (Supplementary file 2). The core search terms considered in all databases were anencephaly, “neural tube defects”, newborns/live births/stillbirths, Africa, and other related terms.

### Inclusion/exclusion criteria and study selection

Studies were eligible for inclusion if they reported the birth prevalence (live births and/or stillbirths) of anencephaly in Africa, studies in any setting, published and unpublished full-text studies in any period, studies reported in the English language, and study designs that reports the birth prevalence/incidence. All newborns with visible congenital neural tube anomaly/defect, anencephaly, identified through clinical evaluation (gross visual examination) by physicians, and/or diagnosed based on ICD-10 classifications, at the time or following the delivery after the twenty-eight weeks of gestation were considered anencephaly cases. Meanwhile, we excluded anonymous reports, conferences, editorials, case reports, and studies without full access (after contacting the author two times through email) from the review. Moreover, studies that did not contain appropriate prevalence data, for instance, review papers or studies providing only calculated estimates (without reporting appropriate numerator/denominator) were excluded. The Endnote Version 7.2 Software was used to import citations following the search of the databases. The three authors (MO, AG, and AA), independent of each other, selected all articles.

### Study validity and data abstraction

We used the JBI quality appraisal checklist to examine the risk of bias in each study^36^. The checklist of studies reporting the prevalence data was considered to evaluate studies (it contains nine items) (Supplementary file 3). Remarkably, two authors (MO, AG) independently evaluate the quality of each study using the template. The discrepancies between authors that arise during examining the quality were solved based on discussions and/or by taking the average score of the two authors.

After including the eligible articles, three authors (AG, AA, and MO) extracted all essential qualitative and quantitative data independently using a standardized, pre-specified, data abstraction template. The data abstraction format included main author, study design, publication year, sample size, study country, study setting, study duration, period prevalence, folic acid fortification policy, and birth prevalence (birth outcome: live births only, and both live births and stillbirths) of anencephaly. In this review, all studies’ prevalence reports in the different denominators have been converted into per hundred births to maintain uniformity and we used per hundred estimates to report the findings. This was calculated directly from data when the appropriate number of cases and denominator were given.

### Statistical analyses and meta-analyses

After extraction of the data in Microsoft Excel and we exported it into STATA Version 14 Statistical Software for further analyses. In addition to the main analysis, we estimated the median value and interquartile range for all included studies. The heterogeneity between the studies was examined using the Cochran Q test and I^2^ test statistics^37^. Due to the significant heterogeneity (*P* value < 0.001), it was decided that estimating a weighted average (via the random-effect meta-analysis model) was the best approach to obtaining the pooled prevalence estimates^38,39^. Furthermore, meta-analyses were carried out separately for each sub-group: birth prevalence based on the study country, study design, birth outcome, period prevalence, and folic acid fortification status. In subgroup analysis, if significant heterogeneity across the sub-groups was detected, the Der Simonian and Laird’s (D + L) pooled prevalence method was considered because it is more conservative (can lead to reliable estimates) than the inverse variance method (I–V). The Forest plot was used to display the presence of heterogeneity among the studies. Meta-regression analysis, sensitivity analysis, time-trend analysis, and meta-cumulative analysis were performed as well. To assess the publication bias, funnel plot asymmetry was used. Essentially, the trim and fill analyses were considered to mitigate the publication bias.

## Supplementary Information

Supplementary Information.

## Data Availability

The data sets used and/or analyzed during the current systematic review and meta-analysis are available from the corresponding author on reasonable request.
